# Thyroid Signaling Biomarkers in Female Symptomatic Hypothyroid Patients on Liothyronine versus Levothyroxine Monotherapy: A Randomized Crossover Trial

**DOI:** 10.1155/2022/6423023

**Published:** 2022-05-04

**Authors:** Betty Ann Bjerkreim, Sara Salehi Hammerstad, Hanne Løvdal Gulseth, Tore Julsrud Berg, Sindre Lee-Ødegård, Erik Fink Eriksen

**Affiliations:** ^1^Department of Endocrinology, Morbid Obesity and Preventive Medicine, Oslo University Hospital, Oslo, Norway; ^2^Institute of Clinical Medicine, Faculty of Medicine, University of Oslo, Oslo, Norway; ^3^Pilestredet Park Specialist Center, Oslo, Norway; ^4^Department of Pediatrics, Oslo University Hospital, Oslo, Norway; ^5^Department of Chronic Diseases and Ageing, Norwegian Institute of Public Health, Oslo, Norway; ^6^Department of Transplantation, Oslo University Hospital, Oslo, Norway; ^7^The Faculty of Dentistry, University of Oslo, Oslo, Norway

## Abstract

**Background:**

Levels of thyroid-stimulating hormone (TSH) are believed to reflect degree of disease in patients with hypothyroidism, and normalization of levels is the treatment goal. However, despite adequate levels of TSH after starting levothyroxine (LT4) therapy, 5–10% of hypothyroid patients complain of persisting symptoms with a significant negative impact on quality of life. This indicates that TSH is not an optimal indicator of intracellular thyroid hormone effects in all patients. Our aim was to investigate different effects of LT3 and LT4 monotherapy on other biomarkers of the thyroid signaling pathway, in addition to adverse effects, in patients with residual hypothyroid symptoms.

**Methods:**

Fifty-nine female hypothyroid patients, with residual symptoms on LT4 monotherapy or LT4/liothyronine (LT3) combination therapy, were randomly assigned in a non-blinded crossover study and received LT4 or LT3 monotherapy for 12 weeks each. Measurements, including serum analysis of a number of biochemical and hormonal parameters, were obtained at the baseline visit and after both treatment periods.

**Results:**

Free thyroxine (FT4) was higher in the LT4 group, while free triiodothyronine (FT3) was higher in the LT3 group. The levels of reverse triiodothyronine (rT3) decreased after LT3 treatment compared with LT4 treatment. Both low-density lipoprotein (LDL) and total cholesterol levels were reduced, while sex hormone-binding globulin (SHBG) increased after LT3 treatment compared with LT4 treatment. The median TSH levels for both treatment groups were within the reference range, however, lower in the LT4 group than in the LT3 group. We did not find any differences in pro-B-type natriuretic peptide (NT pro-BNP), handgrip strength, bone turnover markers, or adverse events between the two treatment groups.

**Conclusion:**

We have demonstrated that FT4, FT3, rT3, cholesterol, and SHBG show significantly different values on LT4 treatment compared with LT3 treatment in women with hypothyroidism and residual symptoms despite normal TSH levels. No differences in general or bone-specific adverse effects were demonstrated. This trial is registered with NCT03627611 in May 2018.

## 1. Introduction 

The thyroid gland produces thyroxine (T4) and triiodothyronine (T3). T4 is considered a prohormone and is converted to T3 by deiodinases to exert biological effects at the tissue level by binding to intracellular nuclear T3 receptors [1]. Serum levels of thyroid-stimulating hormone (TSH) are believed to reflect true intracellular effects of thyroid hormones in peripheral tissues and cells and therefore represent the usual treatment target in patients with hypothyroidism. Despite optimal substitution therapy with levothyroxine (LT4) evaluated by serum TSH levels, still 5–10% of hypothyroid patients exhibit residual hypothyroid symptoms with a major negative impact on quality of life [2]. The reason for this incongruity is unknown. Studies in rats have, however, suggested that LT4 combination therapy with liothyronine (LT3) resulted in more physiological tissue levels of T3 than LT4 alone [3]. Thus, whether TSH values within the normal range reflect adequate intracellular T3 levels and effects in all hypothyroid patients is still subject to debate.

The residual hypothyroid symptoms experienced by patients on LT4 with normal TSH values typically include fatigue, memory loss, and cold intolerance, with poor sensitivity and specificity for the condition [4]. The absence of biomarkers reflecting intracellular T3 levels makes an objective assessment of the severity and morbidity of these symptoms difficult. In addition, studies evaluating alternative treatments to LT4 have not been conclusive as to whether there is a need for a supplement with LT3 in hypothyroid patients [5–15]. Despite a predominance of subjective symptoms, changes in objective parameters among hypothyroid patients have also been demonstrated. Hypothyroidism is associated with impaired physical endurance. This is due to a combination of negative effects on striated skeletal muscle [16] and reduced chronotropic and inotropic reserves of the heart [17] combined with reduced diastolic function [18]. Impaired skeletal muscle function is associated with reduced potential for free fatty acid (FFA) utilization in skeletal muscle, which enhances muscular consumption of glycogen, thereby limiting endurance capacity [19].

Thyroid hormones regulate cholesterol and lipoprotein metabolism. Hypothyroidism affects lipid metabolism negatively leading to hypercholesterolemia with reduced low-density lipoprotein (LDL) receptor activity and diminished control of cholesterol biosynthesis [20, 21]. Hypothyroidism is therefore associated with a non-favorable lipid profile. Substitution therapy with thyroid hormone could therapeutically reverse this negative lipid profile [22]. In accordance with this notion, Celi et al. demonstrated different results on body weight and serum cholesterol levels with LT3 monotherapy compared with LT4 monotherapy, with a significantly better metabolic profile after LT3 treatment [23].

We have already reported both primary and secondary objectives of a randomized crossover study on female hypothyroid patients with residual symptoms using LT4 or LT3 monotherapy for 12 weeks, respectively [24, 25]. Since the serum level of TSH may not be the best indicator of optimal thyroid hormone status in peripheral tissues outside the pituitary, we wanted to evaluate other biomarkers of the thyroid signaling pathway, at treatment doses that normalizes serum TSH, as well as general and bone-specific adverse effects.

## 2. Materials and Methods

### 2.1. Study Design and Patients

**Figure 1:**
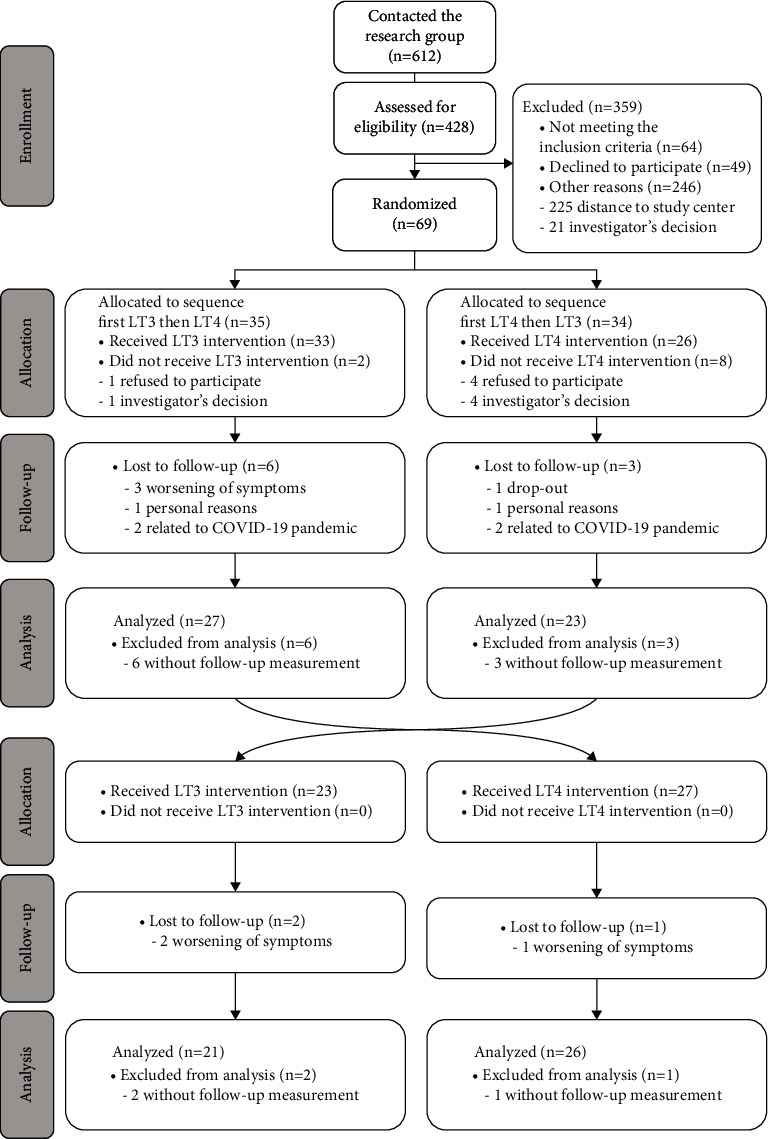
Flow chart showing all subjects approached for the study.

The study design and recruitment process have been described earlier [24]. In brief, 69 female hypothyroid patients aged 18–65 with residual symptoms despite LT4 therapy or combination LT4/LT3 therapy without heart, lung, kidney, or any other endocrinological diseases were randomly assigned by block sizes of 4, 6, or 8 to receive treatment with first LT3 monotherapy or LT4 monotherapy for 12 weeks, before switching to the second treatment for another 12 weeks. However, since ten participants withdrew before the study start, only 59 patients were randomized and received study treatment (Figure 1).

The presence of residual hypothyroid symptoms was ascertained by a ten-question form, where participants had to answer “yes” to at least three questions to be considered eligible for inclusion [24].

If a patient was already on LT3/LT4 combination therapy, LT3 monotherapy, or desiccated thyroid extract before the study start, they had to complete a 4-week run-in period on LT4 monotherapy. We estimated the LT4 dose by calculating 15 *μ*g of LT3 as equal to 50 *μ*g of LT4, based on a pharmacoequivalence study, which showed that the LT4/LT3 equivalence ratio is approximately 3 : 1 [26]. LT3 treatment was started at a dose of one third of the patient's previous LT4 dose. LT4 treatment was maintained at the patient's usual dose. A wash-out period was not included between the two treatment periods due to the shorter half-life of LT3 compared with LT4 and a relatively long treatment period of 12 weeks. After attending the initial study visit, patients were advised to take LT4 treatment once daily, half an hour before breakfast, and LT3 treatment thrice daily, half an hour before, or two hours after, a meal.

Measurements were obtained at a baseline visit and after both 12-week treatment periods of the crossover study. Additionally, thyroid function tests were obtained every four weeks for adjustment of treatment dosage, aiming to achieve TSH levels of 0.1–1.5 mU/L.

The study was carried out between June 2018 and June 2020 at the Department of Endocrinology, Morbid Obesity and Preventive Medicine at Oslo University Hospital.

### 2.2. Biochemistry Assessment and Hormone Analysis

All analyses were performed at the Hormone Laboratory and Department of Medical Biochemistry at Oslo University Hospital. All patients were in a fasting state when blood samples were collected approximately 24 hours after the last LT4 dose and 14 hours after the last LT3 dose.

Thyroid-stimulating hormone (TSH, reference range: 0.5–3.6 mU/L) was measured with noncompetitive immunofluorometric analysis by AutoDELFIA (Wallac Oy, Turku, Finland). Free thyroxine (FT4: 8.0–21.0 pmol/L) was measured with solid-phase time-delayed fluoroimmunoassay with back titration by AutoDELFIA (Wallac Oy, Turku, Finland). Free triiodothyronine (FT3: 2.8–7.0 pmol/L) was measured with competitive electrochemiluminescence immunoassay by Cobas e601 (Roche Diagnostics, Indianapolis, IN, USA). Reverse triiodothyronine (rT3: 0.14–0.54 nmol/L in females) was measured with radioimmunoassay (DIAsource ImmunoAssays, Ottignies-Louvain-la-Neuve, Belgium). Low-density lipoprotein (LDL: 1.9–4.8 mmol/L), high-density lipoprotein (HDL: 1.0–2.7 mmol/L), and total cholesterol (TC: 3.3–6.9 mmol/L) were measured with the enzymatic colorimetric method by Cobas c701/702 (Roche Diagnostics, Indianapolis, IN, USA). Sex hormone-binding globulin (SHBG: 23–100 nmol/L) was determined with a noncompetitive immune luminometric assay by IMMULITE 2000 XPi (Siemens Healthineers, Erlangen, Germany). Pro-B-type natriuretic peptide (NT pro-BNP: <170 ng/L) was determined with electrochemiluminescence immunoassay by Cobas e601 (Roche Diagnostics, Indianapolis, IN, USA). Procollagen type 1 N propeptide (PINP: 11–94 *µ*g/L) and C-terminal telopeptide of type 1 collagen (CTX: ≤0.57 *µ*g/L) were measured with noncompetitive electrochemiluminescence immunoassay by Cobas e601 (Roche Diagnostics, Indianapolis, IN, USA).

### 2.3. Handgrip Strength

To test muscle endurance, handgrip strength was measured using a Jamar Hydraulic Hand Dynamometer (Sammons Preston, Bolingbrook, IL, USA) with patients seated, their elbow by their side and flexed to right angle, and in a neutral wrist position. The calculation of the mean of three measurements of grip strength for each hand was made, and the results were displayed as left or right regardless of hand dominance.

### 2.4. General Adverse Events

Patients were asked to immediately report any occurring discomfort or unfortunate events. In addition, study personnel specifically asked about adverse events at each test visit.

### 2.5. Sample Size and Power Calculations

The calculation of sample size has been described earlier and was based on temperature data since the main outcome in our randomized crossover study was dermal skin temperature and activation of brown adipose tissue [24]. Sixty patients were set as the recruitment target.

### 2.6. Statistical Analyses

All statistical analyses were performed using IBM SPSS Statistics for Windows (Version 26; IBM Corp., Armonk, N.Y., USA) and R (Version 4.0.2; R Core Team, 2016) using the R packages car (Version 3.0.8) and Ime4 (Version 1.1.23). The R packages ggplot2 (Version 3.3.2) and patchwork (Version 1.1.0) were used for visualizations. Statistical significance was defined as two-tailed *P* < 0.05. Data were analyzed by one-way ANOVA and paired *t* tests for variables with normal distribution, Wilcoxon signed-rank test, and Mann–Whitney *U* test for variables without normal distribution, and chi-square test for categorical variables. A mixed-methods model was used for the linear regression analysis, adjusting for period and treatment sequence (crossover effect), and included patients as the random factor. All variables were tested for normality by quantile-quantile (QQ) plots and histogram.

## 3. Results

### 3.1. Characteristics of the Patients

Baseline characteristics (Table 1) have been presented earlier [24] and were similar between patients allocated to the different treatment sequences. In brief, the mean age was 42.9 years ± 9.7 and mean duration of LT4 substitution therapy was 10.6 years ± 7.0, while the median TSH level was 0.64 mU/L (IQR 0.26–1.60) at inclusion. Forty-seven of the 59 included patients completed both treatment periods with LT4 or LT3 for 12 weeks each (Figure 1).

### 3.2. Serum Levels of Thyroid Hormones

The median TSH level was 1.33 mU/L (IQR 0.47–2.26) after 12 weeks on LT3 therapy and 0.61 mU/L (0.25–1.20) after LT4 therapy (*P*=0.018) (Table 2). As expected, we found a significant difference in levels of FT4 (Table 2). FT3 levels were within the reference ranges for both treatment groups, however, with a higher median value after 12 weeks on LT3 compared with LT4 (Table 2).

### 3.3. Serum Reverse T3

Serum levels of rT3 were much lower after 12 weeks of LT3 compared with 12 weeks of LT4 treatment (median 0.03 nmol/L (IQR 0.03–0.05) vs. 0.46 nmol/L (0.38–0.58); *P* < 0.001) (Table 2).

### 3.4. Serum Cholesterol

**Figure 2:**
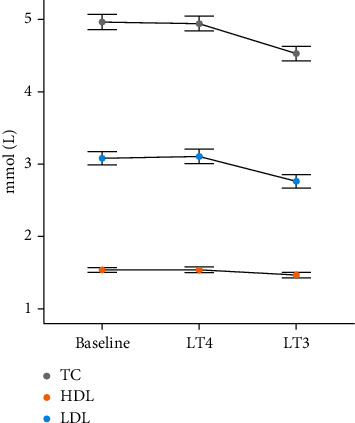
A multiple line graph showing mean (±95% confidence intervals) serum levels of total cholesterol (TC), HDL cholesterol, and LD cholesterol in mmol/L measured at the baseline visit and after both treatment periods with LT4 and LT3.

Twelve weeks of treatment with LT3 reduced levels of both total cholesterol and LDL cholesterol significantly compared with LT4 (Table 2 and Figure 2). The ratio of LDL to HDL cholesterol also decreased after 12 weeks of LT3 treatment compared with LT4 treatment (2.0 mmol/L (1.0–2.9) vs. 2.1 mmol/L (1.1–3.1); *P*=0.026) (Table 2 and Figure 2).

### 3.5. Association between LDL Cholesterol and Parameters of Thyroid Action

**Figure 3:**
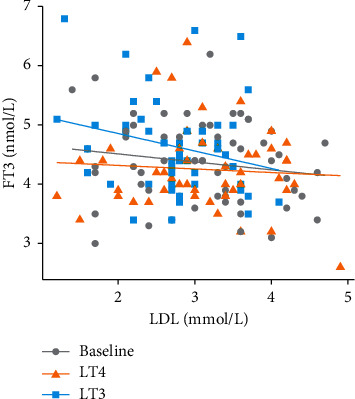
A scatter dot plot showing the association between serum levels of LDL cholesterol and levels of free T3 at baseline (grey line), after LT4 treatment (orange line), and after LT3 treatment (blue line).

For each 1 nmol/L increase in FT3, we observed a reduction of −0.19 mmol/L (95% confidence interval (CI): −0.30, −0.08), *P* < 0.001) of LDL across all conditions. Comparing the conditions, we observed that LT3 treatment resulted in a stronger potential LDL-lowering effect of FT3 than both LT4 treatment (*β* −0.302 (95% CI: −0.442, −0.161); *P* < 0.001) and pre-study treatment at baseline (*β* −0.304 (95% CI: −0.450, −0.158); *P* < 0.001) (Figure 3).

**Figure 4:**
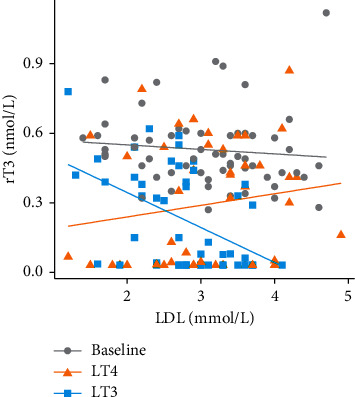
A scatter dot plot showing the association between serum levels of LDL cholesterol and levels of reverse T3 at baseline (grey line), after LT4 treatment (orange line), and after LT3 treatment (blue line).

In addition, when treated with LT3 we observed a negative association between rT3 and LDL concentrations (*β* −1.53 (95% CI: −2.45, −0.62); *P*=0.002) (Figure 4). No associations were observed for rT3 and LDL being on LT4 or pre-study treatment regimes. The association between rT3 and LDL concentrations was moderated, but remained significant after adjustment for FT3 levels (*β* −1.40 (95% CI: −2.35, −0.46), *P*=0.005).

### 3.6. Serum Levels of Pro-B-Type Natriuretic Peptide

Serum levels of NT pro-BNP were similar between the LT3 and LT4 groups (median 52.0 ng/L (IQR 50.0–90.3) vs. 50.0 ng/L (50.0–58.8); *P*=0.057) (Table 2).

### 3.7. Serum Levels of Sex Hormone-Binding Globulin

SHBG levels increased significantly after LT3 treatment compared with LT4 treatment (median 75.0 nmol/L (IQR 49.5–97.0) vs. 56.0 nmol/L (39.3–75.0); *P*=0.001) (Table 2).

### 3.8. Markers of Bone Turnover

Evaluation of bone markers did not show any significant differences between LT3 and LT4 after 12-week treatment periods. However, a significant difference between levels of CTX and PINP was seen after 12 weeks on LT3 compared with baseline. Nevertheless, both these levels remained within the reference ranges (Table 2).

### 3.9. Grip Strength

Fifty-five (93%) of the patients were right-handed, and four (7%) were left-handed. We did not detect any differences in handgrip strength between the two treatments. The values for handgrip strength are presented as left- and right-handgrip strength in Table 3.

### 3.10. General Adverse Events

The number of patients who reported adverse events was similar between the two treatment groups. Seventeen adverse events were reported during LT3 treatment, while 16 adverse events were reported during LT4 treatment (Table 4).

## 4. Discussion

In this study, we have demonstrated that LT3 and LT4 have different effects on a number of biomarkers included in the thyroid hormone signaling pathway in patients with hypothyroidism and residual symptoms despite TSH levels within the reference range. This suggests that evaluation of optimal treatment effects may not be based on TSH levels only, but rather in combination with other biomarkers pertaining to thyroid hormone signaling.

Hypothyroidism is a major cause of secondary dyslipidemia, which may promote the development of atherosclerosis and cause cardiovascular disease. Our results imply that treatment with LT3 restores lipid homeostasis in hypothyroid patients with residual hypothyroid symptoms to a greater extent than LT4, which may be clinically important in reducing number of cardiovascular events in the long term. A 2018 meta-analysis including 65 studies showed that hypothyroid patients on LT4 treatment with normal TSH levels had significantly higher total and LDL cholesterol levels compared with healthy controls [27]. The 2019 European guidelines for the management of dyslipidemias recommend a further reduction in LDL cholesterol levels in both primary prevention and secondary prevention [28].

Epidemiological studies have shown that low SHBG plasma levels are a risk factor for developing cardiovascular disease [29, 30]. In 2005, Alevizaki et al. reported significantly lower SHBG levels in LT4-treated hypothyroid patients compared with healthy controls [31]. In addition, untreated patients receiving LT3 therapy exhibited an increase in plasma SHBG levels [32]. This is consistent with our results with lower levels of SHBG on LT4 treatment compared with LT3 treatment. Since low SHBG levels are associated with an increased risk of cardiovascular events, our findings together with the observed reduction in LDL cholesterol on LT3 treatment might improve cardiovascular status in patients with hypothyroidism. In premenopausal women, however, an increase in SHBG will reduce free sex hormone levels with potentially adverse effects on reproduction and sexual function [33]. This is a potential area of concern, but whether it rises to clinically significant effects remains to be established.

We have demonstrated similar levels of NT pro-BNP in patients treated with LT3 and LT4. Paired with similar blood pressure and resting heart rate between the two study treatments, as previously reported [25], this suggests that LT3 exerts no negative effect on heart function over a period of three months. Indeed, LT3 may exert positive effects on the heart. One study reported reduced levels of NT pro-BNP after six weeks on LT3 monotherapy in patients with chronic heart failure and low-T3 syndrome compared with placebo [34]. However, due to the fact that heart disease was an exclusion criterion in our study, and the relatively young age of the study subjects, the absence of cardiac adverse events on LT3 may have been partially caused by selection bias.

Notably, in addition to initiate gene transcription by binding directly to DNA, thyroid hormone action may also be mediated through cellular compartments besides the nucleus [35]. LT3 monotherapy could potentially lead to the accumulation of T3 hormone in the cytoplasm and activate this alternative pathway of thyroid hormone action. Additionally, it has been reported that thyroid hormones may influence cardiac excitability independent of thyroid hormone receptor signaling on the genome [36]. Consequently, elevated FT3 levels may exert negative effects on the cardiovascular system by increasing the risk of atrial fibrillation. Of the subjectively reported adverse events, there was indeed one episode of transient heart palpitations. Despite this, our data suggest that treatment with LT3 monotherapy in hypothyroid patients without established heart disease is a safe choice in the short term. Long-term follow-up is, however, needed.

Serum levels of bone turnover markers reflect remodeling activity in the skeleton, and elevated levels have been shown to be associated with the increased bone loss [37, 38]. The evaluation of bone turnover markers suggested a slight increase in bone turnover on LT3 compared with baseline. However, in absolute terms the differences were small and both CTX and PINP remained in the middle of the reference range after LT3 treatment, indicating no significant negative skeletal impact due to increased bone loss.

Tissues are protected from excess thyroid hormones through the inactivation of T4 to rT3 by deiodinases [1, 39]. It has previously been reported that LT4/LT3 combination therapy results in reduced levels of rT3 [40]. In this study, patients on LT3 monotherapy exhibited pronounced reductions in rT3 to levels way below the reference range. Nevertheless, our results showed TSH levels within the reference range in the LT3-treated group despite reduced rT3. In addition, the 75 percentiles of rT3 in the LT4-treated group were outside the upper reference range. A possible explanation could be that increased rT3 levels in patients on LT4 monotherapy with residual hypothyroid symptoms may indicate increased degradation leading to reduced intracellular T3 levels. This may also have potential implications for LT4/LT3 combination therapy, where ambient LT4 levels, by activating rT3 formation, might actually prevent achieving sufficiently high levels of T3 intracellularly in some patients. Previously, we reported that only a small fraction of patients with persistent hypothyroid symptoms exhibited mutations in the deiodinase gene [25]. Thus, increased rT3 activity may play a bigger role in the development of low intracellular T3 levels than reduced deiodinase activity in this group of patients. However, since downregulation of peripheral deiodinases is considered a beneficial mechanism for saving energy, decreased levels of rT3 may reflect reduced ability to locally modify thyroid hormone bioactivity during serious illness or fasting. Consequently, LT3 monotherapy may diminish the thyroid homeostasis that normally ensures the absence of excessive levels of thyroid hormones and increase the risk for thyroid storm in precipitating situations.

FT3 levels were higher after LT3 treatment. This may indicate either that conversion of T4 to T3 by deiodinases is insufficient in some patients with hypothyroidism or that intracellular degradation of T3 is enhanced after LT4 monotherapy or LT4/LT3 combination therapy. Thus, administration of extra LT3 seems necessary in some patients with hypothyroidism. The American Thyroid Association Guidelines from 2014 suggest that the recommendation of not using LT3 monotherapy in the treatment of hypothyroidism may change in the future if long-term studies on sustained-release LT3 become available [41].

## 5. Conclusion

Our study demonstrated an improvement in cardiovascular risk factors such as SHBG and total and LDL cholesterol in patients with residual hypothyroid symptoms when treated with LT3 compared with LT4 without differences in NT pro-BNP levels. Also, LT3 did not increase bone turnover. Moreover, we consider the increase in rT3 levels on LT4 treatment an important factor leading to reduced intracellular levels of T3 and residual hypothyroid symptoms despite normal peripheral TSH values. However, future studies are needed to assess the efficacy and safety of long-term LT3 monotherapy.

## Figures and Tables

**Table 1:** Baseline characteristics.

		Prior to randomization (*n* = 59)	Allocated to first LT4 and then LT3 (*n* = 27)	Allocated to first LT3 and then LT4 (*n* = 32)	*P* Value
Age at inclusion (years)		42.9 ± 9.7	42.8 ± 8.6	42.9 ± 10.7	0.991
Age at hypothyroidism diagnosis (years)		30.6 ± 10.2	29.8 ± 8.6	31.2 ± 11.4	0.590
Duration of substitution monotherapy LT4 (years)		10.6 ± 7.0	10.9 ± 7.3	10.3 ± 6.8	0.778

Type of therapy at inclusion					
	LT4 monotherapy	46 (78.0)	20 (74.1)	26 (81.3)	0.513
	LT4/LT3 combination therapy	12 (20.3)	7 (25.9)	5 (15.5)	0.728
	Thyroid extract	1 (1.7)	0 (0)	1 (3.1)	1.000

Etiology of hypothyroidism					
	Autoimmune/idiopathic	56 (94.9)	27 (100%)	29 (90.6)	0.299
	Postsurgical	2 (3.4)	0 (0)	2 (6.3)	0.549
	Radioiodine	1 (1.7)	0 (0)	1 (3.1)	1.000

Body mass index (kg/m^2^)		28.1 ± 5.6	28.5 ± 5.9	27.8 ± 5.5	0.624
Resting heart rate (beats/minute)		64.7 ± 11.2	67.4 ± 12.1	62.4 ± 9.9	0.084
Systolic blood pressure (mmHg)		118.0 ± 13.7	115.5 ± 13.2	120.2 ± 13.9	0.191
Diastolic blood pressure (mmHg)		78.3 ± 7.9	77.2 ± 8.7	79.4 ± 7.0	0.282
TSH (mU/L)		0.64 (0.26–1.60)	0.82 (0.28–1.50)	0.63 (0.25–1.9)	0.767
FT4 (pmol/L)		16.8 (14.7–19.0)	17.0 (14.2–20.0)	16.4 (14.8–18.0)	0.970
FT3 (pmol/L)		4.4 (3.8–4.9)	4.4 (3.8–4.8)	4.4 (3.8–5.0)	0.664
Positive TPO-ab^1^		31 (52.5)	17 (63.0)	14 (43.8)	0.226

Residual hypothyroid symptoms					
	Fatigue	57 (96.6)	26 (96.3)	31 (96.6)	1.000
	Cold intolerance	54 (91,5)	26 (96.3)	28 (87.5)	0.460
	Cognitive disturbances	48 (81.4)	24 (88.9)	24 (75.0)	0.303
	Emotional disturbances	38 (64.4)	16 (59.3)	22 (68.8)	0.627

Data are presented as mean ± SD or number (%) or median (interquartile range: 25–75%) as appropriate. TSH: thyroid-stimulating hormone (ref. range 0.5–3.6), FT4: free thyroxine (8.0–21.0), FT3: free triiodothyronine (2.8–7.0), TPO-ab: thyroid peroxidase antibodies. ^1^ Cutoff value for positive TPO-ab was 35 kIU/l.

**Table 2:** Biochemical and hormonal parameters reflecting thyroid hormone signaling.

	Baseline (*n* = 59)	After 12 weeks on LT4 (*n* = 48)		After 12 weeks on LT3 (*n* = 48)		LT4 vs. LT3 (*n* = 47)
	Result	Result	*P* Value	Result	*P* Value	*P* Value
TSH (mU/L)	0.64 (0.26–1.60)	0.61 (0.25–1.20)	0.155	1.33 (0.47–2.26)	0.232	0.018
FT4 (pmol/L)	16.8 (14.7–19.0)	16.0 (14.4–17.3)	0.226	3.1 (3.1–4.0)	<0.001	<0.001
FT3 (pmol/L)	4.4 (3.8–4.9)	4.2 (3.9–4.6)	0.520	4.6 (4.0–5.0)	0.033	0.008
SHBG (nmol/L)	59.0 (40.0–83.0)	56.0 (39.3–75.0)	0.888	75.0 (49.5–97.0)	<0.001	0.001
rT3 (nmol/L)	0.50 (0.43–0.59)	0.46 (0.38–0.58)	0.029	0.03 (0.03–0.05)	<0.001	<0.001
TC (mmol/L)	5.1 (4.1–5.7)	5.1 (4.3–5.7)	0.352	4.6 (3.9–5.6)	<0.001	<0.001
LDL (mmol/L)	3.2 (2.4–3.6)	3.1 (2.6–3.7)	0.903	2.8 (2.3–3.3)	<0.001	<0.001
HDL (mmol/L)	1.5 (1.3–1.7)	1.5 (1.3–1.7)	0.865	1.5 (1.2–1.7)	0.003	0.005
NT pro-BNP (ng/L)	50.0 (50.0–63.0)	50.0 (50.0–58.8)	0.696	52.0 (50.0–90.3)	0.188	0.057
CTX (*µ*g/L)	0.36 (0.27–0.45)	0.34 (0.24–0.46)	0.623	0.38 (0.27–0.48)	0.009	0.140
PINP (*µ*g/L)	46.7 (38.7–63.7)	50.6 (40.3–71.3)	0.087	50.9 (42.2–68.8)	0.001	0.200

Data are presented as median (interquartile range: 25–75%). TSH: thyroid-stimulating hormone (ref. range 0.5–3.6), FT4: free thyroxine (8.0–21.0), FT3: free triiodothyronine (2.8–7.0), SHBG: sex hormone-binding globulin (23–100), rT3: reverse triiodothyronine (0.14–0.54), TC: total cholesterol (3.3–6.9), LDL: low-density lipoprotein (1.9–4.8), HDL: high-density lipoprotein (1.0–2.7), NT pro-BNP: pro-B-type natriuretic peptide (<170), CTX: C-terminal telopeptide of type 1 collagen (≤0.57), PINP: procollagen type 1 N propeptide (11–94).

**Table 3:** Handgrip strength.

	Baseline (*n* = 59)	After 12 weeks on LT4 (*n* = 49)		After 12 weeks on LT3 (*n* = 48)		LT4 vs. LT3
	Result	Result	*P* Value	Result	*P* Value	*P* Value
Right hand (kilogram)	31.3 ± 5.6	32.0 ± 5.0	0.352	31.4 ± 5.3	0.802	0.307
Left hand (kilogram)	30.4 ± 4.6	30.9 ± 5.1	0.712	30.5 ± 4.5	0.581	0.476

Data are presented as mean ± SD.

**Table 4:** Number and specification of adverse events in the two treatment groups.

Type of adverse event	Number of patients reporting	On LT4 treatment	On LT3 treatment
Cold intolerance	1		1
Myalgia in the neck	1	1	
Bradycardia	1	1	
Ear infection	1	1	
Heart palpitations	1		1
Insomnia	2	1	1
Menstrual disturbances	1		1
Gastroenteritis	4	2	2
Pneumonia	1	1	
Upper respiratory tract infection	8	3	5
Tonsillitis	3	3	
Urticaria	1	1	
Inflammation of hand ligaments	1		1
Iron deficiency anemia	1	1	
Headache	1		1
Tinnitus	1		1
Rib fracture	1	1	
Feeling of high pressure behind the eyes	1		1
Feeling of increased hunger	1		1
Benign paroxysmal positional vertigo	1		1

## Data Availability

The data used to support the findings of this study are available from the corresponding author upon request.
